# Posterior vitreous detachment *in absentia*

**DOI:** 10.1038/s41433-022-02248-3

**Published:** 2022-09-22

**Authors:** Mel J. Maranian, Martin P. Snead

**Affiliations:** Vitreoretinal Research Group, John van Geest Centre for Brain Repair, University of Cambridge, Forvie Site, Robinson Way, Cambridge, CB2 0PY UK

**Keywords:** Pathogenesis, Mechanisms of disease

The relationship between the vitreous body, posterior vitreous detachment (PVD) and retina has been the focus of much research for over 100 years [1–10] and references therein] and yet in spite of such prolonged investigation, there is still much that is not understood, particularly:

1. The changes preceding and initiating uncomplicated (or “physiological”) separation of the posterior hyaloid membrane (PHM) from the retina and,
2. The pathological variations of this process that influence so many of the vitreoretinal disorders dealt with today and thereby their management.

Historically, much has been theorised on the combined biological changes of syneresis and synchisis leading in some ill-defined manner to weakening adhesion at the vitreoretinal interface, inducing the outer vitreous cortex to separate from the inner limiting membrane of the retina. In contrast, more recent research [11–14] has demonstrated that PVD is not simply an inevitable consequence of age related syneresis but more a distinct immunohistochemically confirmed separation of the posterior hyaloid membrane (PHM) from the retinal surface and that separation of this type IV collagen basement membrane can still occur in individuals in whom all cortical gel (syneretic or otherwise) has been previously removed during by vitrectomy surgery.

## Case study

The patient (a low myope [OD −3.25DS, OS −2.75DS]) was referred aged 32 with a serous macular detachment linked to an optic dis pit and vision reduced to counting fingers (CF). The vitreous was attached and the patient underwent pars planar vitrectomy, endolaser and gas tamponade which was not successful in resolving the serous detachment and her vision remained at CF. After further consultation the patient opted for a further attempt at pars planar vitrectomy, endolaser and gas tamponade with a different surgeon, but with the same result and failure to close the link with persistent serous macula detachment.

A year elapsed without change and the patient requested a third attempt at vitrectomy, gas tamponade and endolaser. On this occasion, the surgery was successful in resolving the macular detachment and surprisingly her vision improved from CF to 6/9.

Her crystalline lens remained clear for a further 10 years until she required cataract surgery which was uneventful and restored her vision to 6/9 which she retained for a further 10 years.

Twenty years after her three previous vitrectomy procedures she re-presented with temporal photopsia, a sudden onset of new floaters and a shadow in her vision. Examination revealed a detached PHM, including a Weiss ring and an inferior rhegmatogenous retinal detachment with a single horseshoe tear. The detachment did not involve any of the area previously associated with the optic disc pit and was successfully repaired with a combined vitrectomy and buckle procedure and has remained stable for 2 years with a visual acuity of 6/12.

This case highlights the curious but well recognised phenomenon that “true” PVD (separation of the PHM) can occur independently from, and in the absence of, the vitreous body—PVD *in absentia*.

When it occurs, PVD is usually benign causing few or minor symptoms in most individuals but it can also be the precursor to more serious secondary pathological conditions including but not exclusively, retinal tears, retinal detachment, cellophane maculopathy, vitreomacular traction syndrome and macular hole.

**Fig. 1:**
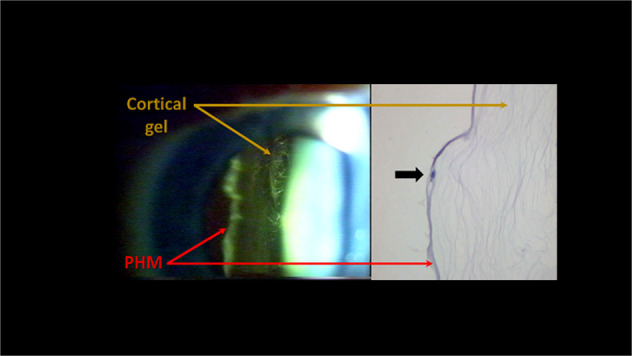
Slit-lamp and light microscopy images of detached posterior hyaloid membrane, showing distinct crinkled and glossy appearance and presence of integral laminocytes (black arrow). Reproduced and adapted with permission from ref. [12].

Research over the last 20 years [11–13] has identified the laminocyte as the novel cell population integral to the detached PHM in both benign PVD and PVD associated with vitreoretinal pathology (Fig. 1). Laminocytes in benign physiological PVD are typically most densely populated encircling the peri-papillary PHM (accounting for the structure commonly referred to as the Weiss ring) but intriguingly are also distributed less densely across the entire detached PHM. Laminocyte distribution within the detached PHM may be associated with focal contracture lending support to the hypothesis that they play an active cellular role in induction of PVD. In PVD associated with vitreoretinal pathologies, laminocytes are typically upregulated and may be associated with marked contracture and even splitting or re-duplication of the PHM [15, 16].

**Fig. 2:**
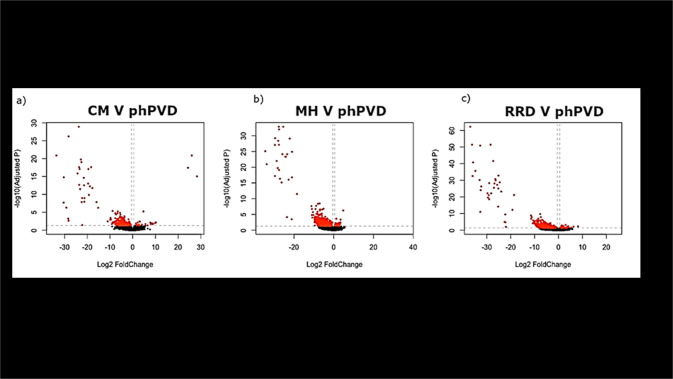
Volcano plot representing DEGs between physiological PVD and PVD associated with secondary pathologies. Log2 FC indicates the mean expression level for each gene, with each dot representing one gene. Red dots are indicative of statistically significant DEGs, upregulated in the pathological groups **a** CM cellophane maculopathy, **b** MH macular hole, **c** RRD rhegmatogenous retinal detachment (adjusted *p* < 0.05) and log2FC < 0.5) (unpublished data).

The cellular factors differentiating benign (physiological) from pathological PVD remain poorly understood. Our current research is investigating the baseline functional transcriptome characteristics of laminocytes within the PHM associated with uncomplicated, physiological PVD when compared with PVD in association with pathological vitreoretinopathies such as cellophane maculopathy, macular hole and retinal detachment using RNA sequencing (RNA-Seq). PHM samples from patients with either uncomplicated (physiological) or pathological PVD collected during surgery have been processed for RNA extraction, library preparation and next generation sequencing. Provisional differential gene expression analysis shows significantly marked differential of expressed genes (DEG) in all pairwise analyses with the overwhelming majority of the DEGs upregulated in each of the pathological PVD groups (Fig. 2).

Gene ontology enrichment analysis is now underway to identify the biological processes and molecular functions that may be over (or under) represented in derived DEG sets in conjunction with a secondary batch of RNA-Seq on fresh and historical tissue samples being analysed using spatial transcriptomics. This innovative technology permits the measurement of gene transcripts “in situ” at distinct spatial locations in the PHM at near-cellular resolution, with the potential to provide a more comprehensive and unique transcriptomic profile of the laminocyte than can be obtained from bulk RNA-Seq alone. Analyses of these singular and combined approaches should provide new insights on a near single cell basis differentiating physiological from pathological PVD.
